# The psychometric properties of the Childhood Health Assessment Questionnaire (CHAQ) in children with cerebral palsy

**DOI:** 10.1186/s12883-018-1154-9

**Published:** 2018-09-20

**Authors:** Soojung Chae, Eun-Young Park, Yoo-Im Choi

**Affiliations:** 1 Department of Secondary Special Education, College of Education, Jeonju University, 1200 3-ga, Hyoja-dong, Wansan-gu, Jeonju, 560-759 South Korea; 2 Department of Occupational Therapy, School of Medicine and Institute for Health Improvement, Wonkwang University, Iksan, South Korea

**Keywords:** Children with cerebral palsy, Childhood health assessment questionnaire, Health-related quality of life, Rasch analysis

## Abstract

**Background:**

The evaluation of children with cerebral palsy (CP) focuses on activity level measurement to examine the effect of health-care interventions on their physical functioning in the home, school, and community settings. This study aimed to identify the psychometric properties of the Korean version of the Childhood Health Assessment Questionnaire (CHAQ) by applying the Rasch model. The use of the Rasch model has an advantage in that item characteristic curve estimation is not affected by the characteristics of subject groups.

**Methods:**

Data were collected from 65 children with CP aged 75–190 months using the Korean version of the CHAQ. Response data were analyzed according to the Rasch model, and item fitness and difficulty and the appropriateness and reliability of the rating scale were evaluated.

**Results:**

Among the 30 items of the Korean version of the CHAQ, two items (nail-cutting and opening a bottle cap that was already opened) were shown to be misfit items with low fitness. The analysis results for item difficulty indicated the requirement for modification of item difficulty, pointing out the need for the addition of question items with both higher and lower difficulty. The use of 4-point rating scale in the evaluation questionnaire was shown to be appropriate. With respect to analysis outcomes, the subjects’ separation reliability value and separation index were 0.97 and 5.92, respectively. In contrast, the separation reliability value and separation index for the question items were 0.95 and 4.51, respectively.

**Conclusions:**

The results of this study suggest the need for the modification of item fitness and difficulty. The psychometric properties of the Korean version of the CHAQ were identified using the item response theory-based Rasch analysis.

## Background

Cerebral palsy (CP) is a permanent and nonprogressive developmental disability. Despite medical treatment and rehabilitation, various motor limitations associated with CP may reduce functionality and affect skills required for the performance of activities of daily living (ADLs) [1]. Premature mortality in children with CP is rapidly decreasing, and most of them survive until adulthood [2, 3]. The acquisition of high-level information about the functional status of children with CP has progressively become imperative [4, 5]. However, information on health-related quality of life (HRQL) in children with CP, which could provide critical perspectives in preparation for the future of these children, remains lacking [6].

Unlike in the past, the evaluation of children with CP focuses on activity level measurement to examine the effect of health-care interventions on their physical functioning in the home, school, and community settings [7, 8]. The International Classification of Functioning (ICF) has provided a framework for the collection of data on aspects of activity limitation and impairment, urging the exploration of the correlation between activity limitation and impairment. The ICF defines activity as the execution of any specific task by an individual [9]. When assessing disability, all relevant circumstances must be taken into account, and the extent to which individuals with disabilities can perform essential functions or major life activities should be measured.

Recently, evaluation has included the role of childhood or how children with disabilities feel in the course of solving obstacles that they face [10–12]. HRQL, a concept pertaining to aspects of life quality that are directly associated with health status, has been assessed [13, 14]. A HRQL assessment inventory had been developed for the past 10 years, with some general scales of HRQL having already been applied to children with disabilities and used for the evaluation of physical and psychological damage [15–18]. Various assessment inventories have been adapted in Korea, including the Korean version of EuroQol-5 Dimensions, which is designed to assess health status with respect to five areas of HRQL, namely mobility, self-care, usual activities, pain/discomfort, and anxiety/depression. The indices for each area are substituted with the assessment index equation to calculate the subjective HRQL indices [19, 20]. The Korean version of the 12-item Short Form Health Survey developed for the Medical Outcomes Study is also a tool used to measure HRQL and comprises physical and mental component summaries (12 items in total), with a higher score indicating a higher HRQL level. Moreover, the entire inventory was reported to be reliable (Cronbach’s α = 0.810) [21]. The Korean version of the World Health Organization (WHO) Quality of Life Scale, an abbreviated modified version of the WHO Quality of Life Assessment Instrument-100 translated into Korean [22], is a 5-point scale inventory consisting of four domains (physical health, psychological, social, and environmental) for each of 24 facets related to quality of life. A response of “never” and “always” corresponds to a score of 1 point (lowest score) and 5 points (highest score), respectively.

These assessment tools have been used to evaluate HRQL in children with different types of disabilities. Based on the results, various plans have been proposed, and assistance has been recommended and provided. However, general scales for HRQL do not directly address functionality or ADL-related concepts, with only few tools among several scales being suitable for children with CP [23–25]. In particular, there remains a lack of information about HRQL in children with CP in Korea, possibly owing to the absence of feasible tools for HRQL measurement.

Recently, the Childhood Health Assessment Questionnaire (CHAQ), a tool specially developed for the assessment of functional capacity and independence in everyday life, has been utilized in children with CP. The CHAQ is a validated questionnaire comprising specific items used to evaluate juvenile idiopathic arthritis in children and adolescents [26] and has been considerably applied to patients with current mobility restrictions due to other chronic diseases such as pediatric spondyloarthropathies, spina bifida, joint hypermobility syndrome, and systemic lupus erythematosus [27–31]. The CHAQ has already been translated into several languages and used in many countries [32]. In Korea, the CHAQ was adapted by Park [33]; since then, its usefulness for the health-related assessment of children with CP has been reported.

The rapidly growing development of comprehensive question items to measure functional health status and quality of life in children has left a task of whether measurement of general aspects or specific conditions should be considered in selecting a tool [34–36]. The tool should provide appropriate information, and its psychometric properties could be measured to check its validity. Further, the tool can be selected only when it is practical, reliable, and appropriate and is able to measure change or sensitive to the change [37].

The classical method of scale verification is to confirm construct validity using factor analysis. However, determining the construct validity of the scale by factor analysis is limited because it is not a confirmation at the level of question [38]. The scale verified by factor analysis is occasionally adapted in other cultural regions in the course of its utilization and is applied to other groups with different characteristics from the respondent group participating in scale development. As scales are diversely utilized, an argument emerges from a study on scale development that factor analysis itself cannot accurately evince validity [39]. Therefore, in order to accurately estimate the fitness and difficulty of items derived from factor analysis, attempts to verify them using various statistical methods are required.

Among these attempts, the Rasch model is one of the item response theory models increasingly used as an appropriate research method for the assessment of the appropriateness of item fitness and difficulty [40]. When measuring the ability of a subject using traditional methods, the same subject will attain a higher and lower score if administered with a lower and higher level of test, respectively. In other words, in traditional methods, the characteristics of children with CP could affect ability measurement, possibly influencing the validity analysis of measurement tools. As the psychometric properties of an instrument can vary among different population groups and can be particularly affected by the cultural context, a systematic assessment of psychometric properties is imperative before an instrument can become extensively used within a specific patient population [41].

Therefore, in order to evaluate the psychometric properties of the CHAQ for assessing HRQL in children with CP in Korea, it is necessary to use data obtained from Korean children with CP. An item response theory-based analysis could be utilized to scrutinize question items using the item characteristic curve unique for each item, examine the difficulty and discrimination power of each item, and estimate the real ability of the subject based on analysis results. In addition, the use of the Rasch model has an advantage in that item characteristic curve estimation is not affected by the characteristics of subject groups [42]. Although the suitability of the Korean version of the CHAQ as a tool based on the classical test theory has already been confirmed in a validity testing study [33], attempting to verify the nature of question items by applying the Rasch model based on the item response theory remains essential to accurately evaluate item fitness and difficulty.

Therefore, this study aimed to identify the psychometric properties of the Korean version of the CHAQ in children with CP by applying the Rasch model. In view of the objective of this study, the following specific research questions were raised: First, is the item fitness of the Korean version of the CHAQ appropriate for children with CP? Second, is the item difficulty of the Korean version of the CHAQ appropriate for children with CP? Third, are the response categories of the Korean version of the CHAQ appropriate for children with CP? Fourth, is the Korean version of the CHAQ reliable when used in children with CP?

## Methods

### Subjects

For subject recruitment, the researchers sent letters to professionals at a hospital or community welfare center, asking for referrals of eligible subjects. Initially, 73 children with CP desired to participate in this study. Subsequently, the researchers sent letters describing the details of this study and received 65 informed consent forms for participation from the parent(s) of children with CP. As incentive, gift cards equivalent to approximately 20,000 won each were provided to the subjects’ parent(s) and therapists. School-age children diagnosed with spastic CP who provided parent consent for their participation in the study were included, whereas those who underwent elective dorsal rhizotomy and had spina bifida and associated musculoskeletal disorders such as muscular dystrophy and myopathy were excluded from the study. Evaluation was performed by one physiotherapist and one caregiver per one child with CP for over 6 months of treatment. The general characteristics of children with CP are summarized in Table 1. The mean age of subjects in this study was 113.14 months (standard deviation = 30.03; range, 75–190 months) (Table 1).

**Table 1:** General characteristics of the study subjects

Classification		Number	%
Gender	Male	45	69.2
	Female	20	30.8
Site of Palsy	Quadriplegia	15	23.1
	Triplegia	4	6.2
	Paraplegia	28	43.1
	Hemiplegi	15	23.1
	Missing Data	3	4.6
Type	Spastic	53	81.5
	Athetoid	9	13.8
	Hypotensive	1	1.5
	Combined Type	2	3.1
Gross Motor Function Classification System	Level 1	16	24.6
	Level 2	14	21.5
	Level 3	10	15.4
	Level 4	6	9.2
	Level 5	19	29.2
Total		65	100

### Tools

#### Childhood health assessment questionnaire

The CHAQ is a tool developed to assess health status and HRQL in children and adolescents with juvenile idiopathic arthritis. It has already been applied to children with various types of disabilities to measure their functional capacity and independence in performing ADLs during the previous week at evaluation time point. The CHAQ covers the following eight domains: dressing and grooming, standing, eating, walking, hygiene, hand stretch, catching, and activities. Items in these respective areas are rated on a 4-point rating scale, with score ranging from 0 to 3 points. A score of 0, 1, or 2 points denotes the performance of certain tasks without difficulty, with some difficulty, and with much difficulty, respectively, whereas a score of 3 points suggests inability to execute tasks. Because some questions are not applicable to young children, such item is marked as “not applicable.” A higher score means low functional capacity. The CHAQ includes two visual analogue scales for the evaluation of overall well-being and pain severity. The eight domains are evaluated with the highest score for the detailed items being recorded, and the overall average is interpreted using the CHAQ Disability Index, with 0 point and 3 points indicating the absence of disability and presence of serious physical disability, respectively [32]. In calculating the CHAQ Disability Index, the highest score for the subquestions under each area is selected. A score of at least 2 points is indicated when a child requires some help in performing certain activities, and the average score for each area is considered the value for the CHAQ Disability Index. In this study, we used the CHAQ adapted to the Korean population [33].

#### Gross motor function classification system

The Gross Motor Function Classification System (GMFCS) was used for the evaluation of gross motor function in children with CP. The GMFCS, a tool designed to evaluate motor disorders in children with CP, categorizes these children into the following five levels: level 1 children, or those who can walk without restrictions; level 2 children, or those who walk with some limitations in most settings; level 3 children, or those who may walk without physical assistance but are using handheld crutches, canes, or walkers; level 4 children, or those who can move around with some limitations by themselves using electric-powered wheelchair or other means of transportation in most settings; and level 5 children, or those who have seriously limited ability to move around by themselves even with the use of assistive devices [43].

### Procedures

Measured data from a total of 65 children with CP who provided informed consent for their participation in this study were collected to determine the psychometric properties of the CHAQ. In this study, a secondary analysis of data collected from a structural equation modeling study on factors affecting the participation of children with CP in daily life activities was performed [44]. The GMFCS was used by a physiotherapist with experience in treating children over 6 years, whereas the CHAQ was used by a caregiver.

### Data processing

An infit mean square statistic (MNSQ) value < 0.5 or > 1.7 for each item denoted unacceptable item fitness [42]. The relation between individual attribute scores and item difficulty was analyzed using the distributions of items and subjects, which were included in a graph according to respective individual attribute scores to enable a direct comparison. Because the individual attribute scores and item difficulty were correspondingly converted into a logit scale for a direct comparison, it was possible to evaluate whether the item difficulty was appropriate for the analyzed group. When the ranges of two different distributions were consistent (i.e., similar distribution ranges for item difficulty such that item difficulty measurement could estimate all ranges of individual attribute scores), the distribution was considered sufficient [45].

The rating scale was analyzed using changes in threshold and the fit index of each subject for each rating score. In general, the higher the rating scores, the higher the proficiency estimates and threshold were for subjects who responded to the questionnaire.

In Rasch analysis, the standard error of measurement is calculated based on all proficiency estimates apart from that of the sample group. This is shown as two concepts, namely the subject separation index and the item separation index. A larger value for these two separation indices indicates an accurate functional measurement level [42]. Based on these concepts, changes in separation reliability were drawn through removal of misfit items and subjects who inadequately responded. A total of 14 subjects inadequately responded in this study.

We used WINSTEPS version 3.6 [46] as statistical software to adapt the Korean version of the CHAQ and apply the Rasch model.

## Results

### Item fitness

The estimation results for the fitness of the 30 items of the CHAQ are summarized in Table 2. As shown in Table 2, the infit MNSQ value for items 4 and 23 following estimation of fitness among all items of the CHAQ was > 1.7 and < 0.5, respectively. After estimating the fitness of all items, the result for the fitness of four items among the 30 items of CHAQ indicated an infit MNSQ value of > 1.7, whereas the infit MNSQ value for item 23 was < 0.5 (Table 2).

**Table 2:** Item fit statistics: entry order

Item	Measure	S.E.	Infit		Outfit	
			MNSQ	Z-value	MNSQ	Z-value
1	29.93	2.42	1.03	0.20	0.88	0.00
2	33.73	2.40	0.58	−2.20	0.54	−0.70
3	58.70	2.76	0.84	−0.60	1.00	0.10
4	27.59	2.43	2.28	4.30	1.93	1.40
5	57.20	2.73	1.20	0.80	0.88	−0.20
6	61.83	2.84	1.27	1.10	0.73	−0.60
7	36.62	2.41	1.54	2.20	1.24	0.60
8	64.29	2.90	0.69	−1.20	0.81	−0.30
9	48.85	2.56	1.54	2.10	1.70	1.80
10	48.20	2.55	1.39	1.60	1.05	0.30
11	42.55	2.47	1.70	2.70	1.35	0.90
12	41.94	2.46	0.64	−1.80	0.66	−0.80
13	48.20	2.55	0.64	−1.70	0.50	−1.60
14	57.20	2.73	0.56	−2.10	0.44	−1.90
15	55.73	2.69	0.67	−1.50	0.57	−1.30
16	50.17	2.58	0.61	−1.90	0.54	−1.50
17	58.70	2.76	1.22	0.90	1.49	1.30
18	58.70	2.76	0.80	−0.80	0.61	−1.10
19	56.46	2.71	0.62	−1.70	0.58	−1.30
20	73.40	3.16	0.98	0.00	2.12	1.50
21	64.29	2.90	0.72	−1.10	0.87	−0.10
22	59.47	2.78	0.82	−0.70	0.79	−0.50
23	65.99	2.94	0.47	−2.40	0.79	−0.30
24	62.64	2.85	0.68	−1.30	0.76	−0.50
25	62.64	2.85	0.58	−1.80	0.78	−0.40
26	36.04	2.41	0.93	−0.30	0.78	−0.20
27	36.04	2.41	0.87	−0.50	0.67	−0.50
28	36.62	2.41	0.47	1.90	1.14	0.40
29	32.02	2.39	1.08	0.40	0.98	0.20
30	34.31	2.40	1.43	1.80	1.11	0.40

*MNSQ* Mean Square, *SE* Standard Error

### Item difficulty

**Fig. 1:**
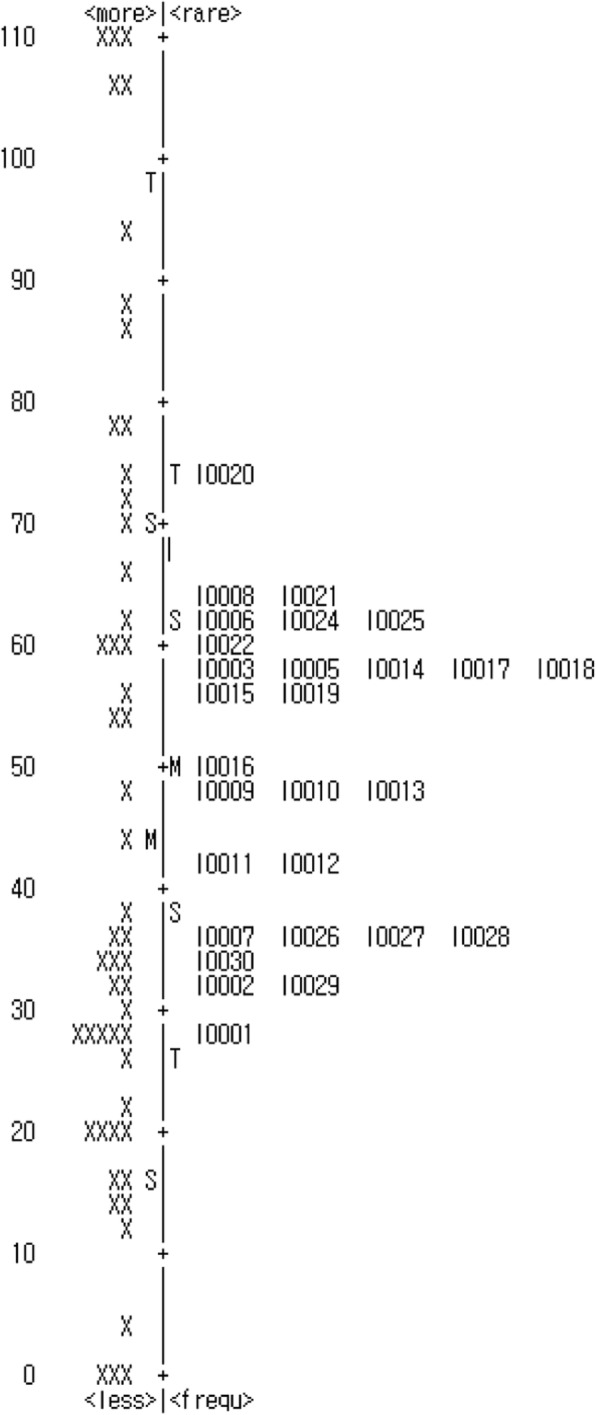
Map of personal proficiencies and item difficulties for the 28 items of the Childhood Health Assessment Questionnaire

In the comparison of individual attribute scores and item difficulty, items 20 and 1 appeared to have the highest and lowest difficulty, respectively. Moreover, proficiency estimates were higher and lower than item difficulty in 22 and 24 subjects, respectively (Fig. 1).

### Rating scale analysis

**Fig. 2:**
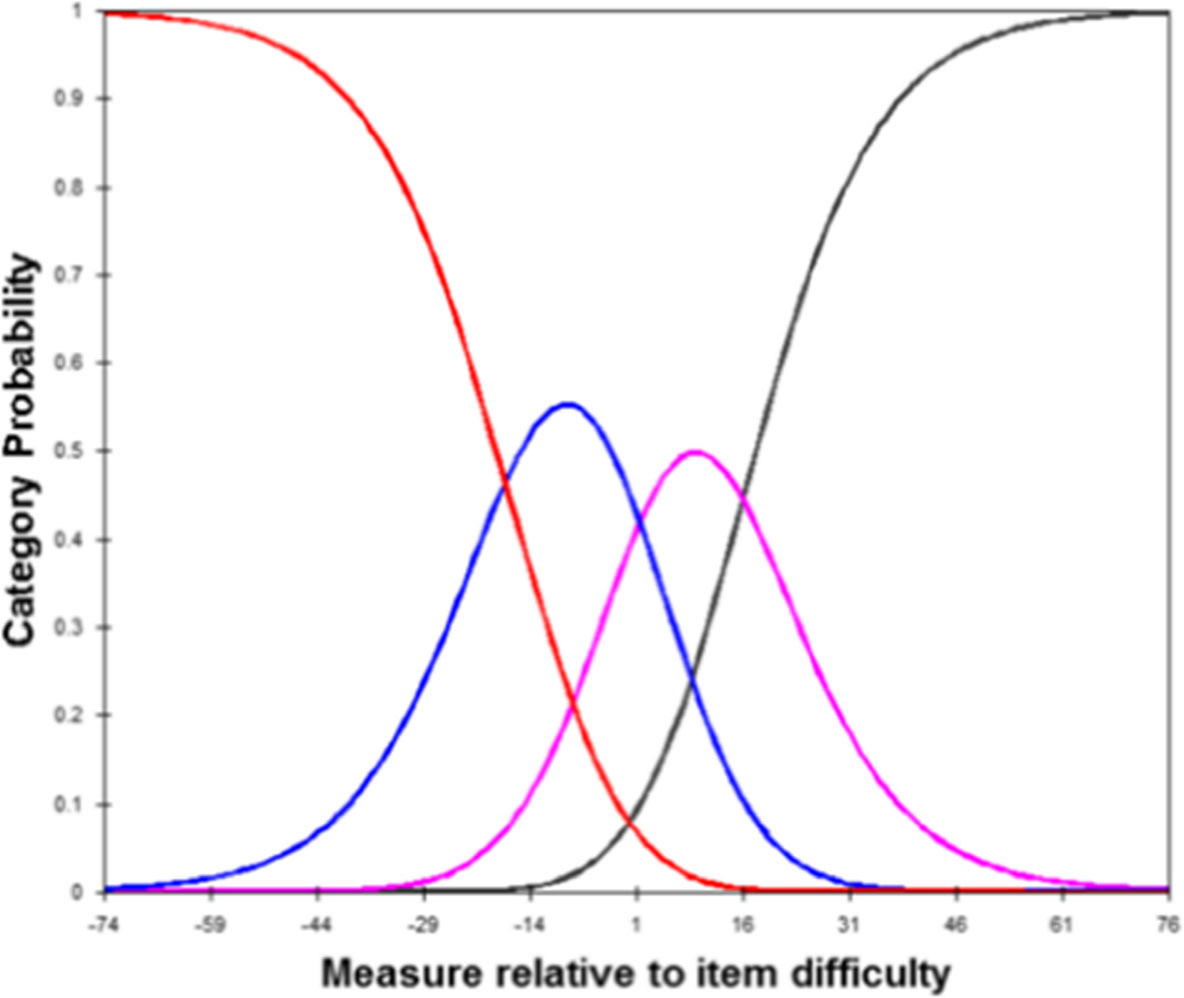
Category probability curve for the Childhood Health Assessment Questionnaire

The estimation results for the 4-point rating scale of the CHAQ are shown in Table 3 and Fig. 2. The analysis results indicated that, for the CHAQ adapted to the Korean population, the increase in scale scores corresponded to an increase in the average proficiency estimate in subjects. Furthermore, the fit index for each scale score provided information on whether the rating scale was properly functioning. The fit index for the individual scale scores showed values ≥1.3 with respect to a value of 1.0, implying that the applicable scale was not properly functioning. The results of the analysis performed showed that there was no misfit scale in the CHAQ. With an increase in each estimate, similar to that in the average proficiency estimate in subjects, the threshold must show an increasing tendency as well. The analysis of the scale threshold showed that the threshold was proportional to the increase in scale scores in all subscales.

**Table 3:** Summary of the rating scale analysis of original 4 point scale

Category Level	Observed Average	Infit MNSQ	Outfit MNSQ	Structure Calibration
0	−33.19	0.92	0.95	None
1	−11.77	1.02	1.01	−17.48
2	8.18	0.87	0.70	1.50
3	33.06	1.31	1.26	15.95

### Separation reliability

The separation reliability of the CHAQ adapted to the Korean population is shown in Table 4.

**Table 4:** Separation Reliability of the Study Subjects and the Items

	Mean	Standard Deviation	Separation Reliability	Separation Index
Subjects	34.0	26.8	.97	5.92
Items	67.3	18.0	.95	4.51

The subjects attained a separation reliability value of 0.97 and separation index of 5.92, whereas the separation reliability value and separation index for the items were 0.95 and 4.51, respectively.

## Discussion

This study aimed to identify the fitness and difficulty of the CHAQ items adapted to the Korean population and verify the appropriateness and reliability of the rating scale in children with CP. To address such objectives, Rasch analysis of 65 subjects was performed using the CHAQ adapted to the Korean population.

When the fitness of the CHAQ items was determined, 2 of 30 items were shown to be misfit items. Item fitness is used to confirm the unidimensional nature of test items and is estimated using the MNSQ value via the utilization of the rating scale model, which could reveal how each item is adequately configured to confirm its unidimensional nature. A high MNSQ value indicates that the item does not have homogeneity with other items within the scale. In contrast, a low value means that the item is redundant with other items [45]. The MNSQ presents two values: the infit index and the outfit index. The infit and outfit indices are standardized, with the standardized value presented as Z value. In the Rasch model, an MNSQ value of 1 represents an ideal value. In this study, each item with an infit index < 0.5 or > 1.7 was regarded as a misfit item in order to determine item fitness [42]. Item 4, which pertained to nail-cutting, had an infit index ≥1.7, whereas item 23, which involved opening a bottle cap that was already opened, had an infit index ≤0.5.

Difficulties associated with the use of the CHAQ adapted to the Korean population were analyzed by comparing individual attribute scores and item difficulty. When the distribution ranges of the individual attribute scores and item difficulty were consistent (i.e., similar distribution ranges for item difficulty such that item difficulty measurement could estimate all ranges of individual attribute scores), the distribution was considered sufficient [45]. The analysis results indicated that the difficulty for item 1 (tying shoe laces and buttoning) was the lowest among the 28 items with low fitness, whereas the difficulty for item 20 (turning the head to see behind the shoulder) was the highest. With respect to item difficulty for the CHAQ adapted to the Korean population, 23.5% and 13.7% of children showed a lower and higher capacity, respectively. A high percentage of floor effect for the measurement tool indicates that the item difficulty is higher than the proficiency estimate assessed using a tool in subjects. Conversely, a high percentage of ceiling effect for the measurement tool indicates that the item difficulty is lower than the proficiency estimate in subjects; hence, it is impossible to assess subjects exhibiting higher proficiency estimates as the item difficulty is too low. In the study of Park [33], the percentage of floor effect was reported to be 4.3–38.6%, with the rate for standing, walking, hand stretching, and catching exceeding 20%. In the case of ceiling effect, the percentage of floor effect was reported to be 1.4–25.7%, with the rate for walking and hygiene exceeding 20%. In the study of Morales et al. [32], the percentage of floor and ceiling effects was reported to be 2.1–26.0% and 30.2–68.8%, respectively. The high percentage of ceiling effect in the study of Morales et al. [32] should be considered, as the proportion of level 1 subjects according to the GMFCS was high (37.5%), and the results of Park’s study [33] should had been affected by the fact that the proportion of level 1 children was 22.2%. Unlike in previous studies that identified item difficulty based on the classical test theory, the subjects’ proficiency estimates and item difficulty in this study were converted into logit interval scales and analyzed, making it possible to overcome the limitations of subjects. Nevertheless, the results of this study showed that there was a need to add items characterized by both high and low difficulty to the CHAQ adapted to the Korean population.

The rating scale to be used for the development of a test should have a clear response level as same as potential variables to be measured and used to produce a test with a rating scale shall have a clear response level. Furthermore, fit indices for each scale score provide information on whether the rating scale is properly functioning. The fit index for the individual scale scores showed values ≥1.5 with respect to a value of 1.0, implying that the applicable scale was not properly functioning and providing possible information on whether the scale scores could be merged later [46, 47]. In the analysis, the 4-point rating scale of the CHAQ adapted to the Korean population was determined to be appropriate. The scale threshold estimate showed a tendency similar to that of the average proficiency estimate in which the scale threshold increased with an increase in scale scores. The scale threshold estimate differs from the average proficiency estimate in subjects in that the former is an estimate calculated by observation frequency based on the sample, whereas the latter is an estimate calculated using the Rasch model [42]. The analysis results showed that the rating scale of the Korean version of the CHAQ is proportional to the increase in scale score, indicating that the response range was appropriate.

The Rasch analysis estimates two types of separation reliability: subject separation reliability and item separation reliability. The Rasch model is able to estimate the concurrent validity through the subject separation reliability and estimate the construct validity through the item separation reliability [48]. The subject separation reliability is the same concept as the conventional reliability, Cronbach’s α [46]. When the CHAQ separation reliability was estimated after excluding the misfit subjects and misfit topics, the subject separation reliability was 0.97, and the separation index was 5.92, whereas the item separation reliability was 0.95, and the separation index was 4.51. These results showed the CHAQ adapted to the Korean population had a high level of reliability.

This study has some limitations. First, it would have been preferable to have more than 65 children with CP as subjects. Further study with a larger sample size is required to increase the power and possibility for generalization of study results. Second, this study only included children with CP aged 75–190 months. This could potentially affect the feasibility to generalize the results to the entire pediatric population with CP. Future studies on infants (0–36 months) and/or young children (36–72 months) with CP should be performed. Lastly, this study did not examine differential item functioning. There may be items that function differently depending on the type of CP; hence, further analysis based on the types of CP is required.

## Conclusions

In this study, the results of assessment performed using the CHAQ adapted to the Korean population were analyzed using the Rasch model to determine the psychometric properties of the CHAQ as a tool for HRQL measurement in children with CP. With the analysis, results for item fitness and difficulty, rating scale analysis, and reliability outcomes were derived. Based on the results, among the items of the CHAQ adapted to the Korean population, two items had low fitness, and modification of item difficulty was required. The rating scale was reliable and appropriate. Moreover, item development and modification were determined to be necessary to augment the usefulness of the CHAQ adapted to the Korean population in assessing HRQL in children with CP. Further studies clarifying the usefulness of tools in assessing quality of life in children with CP should be performed.

## Abbreviations

ADLs: Activities of daily living
CHAQ: Childhood Health Assessment Questionnaire
GMFCS: Gross Motor Function Classification System
HRQL: Health-related quality of life
ICF: International Classification of Functioning
MNSQ: Mean square statistic
WHO: World Health Organization
